# Transcription-Aided Selection (TAS) for Crop Disease Resistance: Strategy and Evidence

**DOI:** 10.3390/ijms252211879

**Published:** 2024-11-05

**Authors:** Jiu Huang, Guangxun Qi, Mei Li, Yue Yu, Erte Zhang, Yuhui Liu

**Affiliations:** 1 Biotechnology Research Institute, Chinese Academy of Agricultural Sciences, Beijing 100081, China; huangjiuhj@163.com (J.H.); limei_caas@163.com (M.L.); tgsxyyy@126.com (Y.Y.); zhanget2764@163.com (E.Z.); 2 Soybean Research Institute, Jilin Academy of Agricultural Sciences, Changchun 130033, China; qiguangxunyl@163.com

**Keywords:** crop disease resistance, transcription-aided selection, SA signaling pathway

## Abstract

A transcription-aided selection (TAS) strategy is proposed in this paper, which utilizes the positive regulatory roles of genes involved in the plant immunity pathways to screen crops with high disease resistance. Increased evidence has demonstrated that upon pathogen attack, the expression of diverse genes involved in salicylic acid (SA)-mediated SAR are differentially expressed and transcriptionally regulated. The paper discusses the molecular mechanisms of the SA signaling pathway, which plays a central role in plant immunity, and identifies differentially expressed genes (DEGs) that could be targeted for transcriptional detection. We have conducted a series of experiments to test the TAS strategy and found that the level of *GmSAGT1* expression is highly correlated with soybean downy mildew (SDM) resistance with a correlation coefficient R^2^ = 0.7981. Using RT-PCR, we screened 2501 soybean germplasms and selected 26 collections with higher levels of both *GmSAGT1* and *GmPR1* (Pathogenesis-related proteins1) gene expression. Twenty-three out of the twenty-six lines were inoculated with *Peronospora manshurica (Pm)* in a greenhouse. Eight showed HR (highly resistant), four were R (resistant), five were MR (moderately resistant), three were S (susceptible), and three were HS (highly susceptible). The correlation coefficient R^2^ between the TAS result and *Pm* inoculation results was 0.7035, indicating a satisfactory consistency. The authors anticipate that TAS provides an effective strategy for screening crops with broad-spectrum and long-lasting resistance.

## 1. Introduction

Plant innate immunity is comprised of two defense systems: pathogen-associated molecular pattern-triggered immunity (PTI) and effector-triggered immunity (ETI) [1,2]. These systems are known as vertical and horizontal resistance, respectively, depending on the presence of a specific interaction between the pathogen and host [3]. Vertical resistance is race-specific, meaning that it only provides protection against certain pathogenic races, while horizontal resistance, which is controlled by multiple genes, offers broad-spectrum and persistent protection [4]. While transgenic crops with insect resistance and herbicide tolerance have been successfully developed and commercialized, transgenic crops with resistance to bacterial or fungal pathogens have not been widely adopted due to a lack of understanding of the complex regulatory networks involved in horizontal resistance [5,6]. Recent studies on SA signal transduction pathways and genome-wide transcriptomic sequencing have provided new insights into this complex system.

SA is a phenolic plant hormone primarily recognized as a defense-related hormone [7,8,9,10,11,12,13]. The initial observations linking SA to plant immunity were made by Raymond F. White in 1979, who noted that the application of aspirin (acetyl-SA) in virus-susceptible tobacco (*Nicotiana tabacum*) provided resistance against tobacco mosaic virus [8]. Additionally, it plays significant roles in alleviating abiotic stresses, such as heat, cold, drought, UV radiation, heavy metals, and osmotic shock [14,15]. However, SA is most renowned for its function in coordinating plant immune responses.

Studies over the past three decades have shown that salicylic acid (SA) signaling plays a central role in plant immunity. The activation or inhibition of diverse defense genes, which are regulated by plant hormone signal transduction, is crucial to the immune response. SA is involved in the activation of defense responses against biotrophic and hemi-biotrophic pathogens [16,17], while jasmonic acid and ethylene are responsible for defense against necrotrophic pathogens [18,19]. Upon pathogen attack, the expression of diverse genes involved in SA-mediated SAR (systemic acquired resistance) responses are differentially expressed and transcriptionally regulated. The SA signaling network (including SA biosynthesis, its regulation and metabolism, and long-distance transportation) has been extensively studied, and genes associated with SA signaling have been identified in various cellular locations (cell membrane, chloroplast, peroxisome, cytosol, and nuclear), all of which play important roles in plant immunity. For a more comprehensive understanding of this topic, readers can refer to reviews written by various authors [17,20,21,22].

### 1.1. Biosynthesis of Salicylic Acid

SA is synthesized in the chloroplast and cytosol in response to pathogen attack. Two major pathways for SA biosynthesis have been identified in plants: the Isochorismate Synthase (ICS) pathway and the Phenylalanine Ammonia-Lyase (PAL) pathway [17,22,23,24]. Both pathways begin with the conversion of chorismic acid to isochorismate, which is then further converted to SA. The chorismate is a product of the Shikimate pathway. The ICS pathway involves the transport of isochorismate from the plastid to the cytosol, while the PAL pathway converts phenylalanine to benzoic acid, which is then converted to SA [22].

An alternative PAL pathway exists in Arabidopsis, where pipecolic acid, Pip, is produced from lysine and subsequently oxidized into N-hydroxypipecolic acid (NHP) in the cytosol [22,25,26]. The PAL pathway is the main route for TMV-induced SA biosynthesis in tobacco [27] and has been shown to contribute to SA biosynthesis in Arabidopsis, rice, and soybean [28,29,30,31]. However, the mechanism by which different plant species preferentially use ICS or PAL or a combination of both is unclear.

Transcription factors SAR-Deficient 1 (SARD1) and CaM-Binding Protein 60 g (CBP60g) have been shown to control the expression of *ICS1* and the accumulation of SA in response to pathogen infection in Arabidopsis. Loss of both SARD1 and CBP60g leads to a block in the induction of ICS1 and SA accumulation. Upon pathogen infection, the CBP60g and SARD1 directly bind to the promoter of *ICS1*, thereby activating its expression [32,33,34]. The same transcription factors also coordinately control the genes involved in NHP biosynthesis. ChIP (Chromatin Immune Precipitation) analysis has shown that SARD1 and CBP60g target not only the genes involved in SA biosynthesis but also those involved in NHP biosynthesis [35,36].

### 1.2. Positively Regulated Genes in SA Signaling

The genes involved in SA signaling that are positively regulated have been extensively studied and summarized based on published data [20,22,37,38,39,40,41]. These genes include calcium ion channel genes (such as calmodulin, CaM and Ca^++^ binding proteins, *CBP*), genes involved in SA and NHP biosynthesis (such as *ICS1*, *PAL*, *SARD1*, and *CBP60g*), the master regulator *NPR1* gene (nonexpressor of pathogenesis-related gene1), genes involved in the oxidative burst and anti-oxidation, and genes coding for pathogenesis-related (PR) proteins.

NPR1 functions as a transcriptional co-activator in SA signaling. In the promoter region of *NPR1*, there are two continue W-box elements with a TGAC core sequence that is overlapped with the SA-responsive element, *as*-1 [42]. Mutations of either *NPR1* or W-box sequences can completely abolish SA-induced SAR and greater susceptibility to hemi-biotrophic pathogens. Currently, it is known that the NPR1 and NPR3/NPR4 are receptors of SA. SA disrupts NPR1–NPR4 interaction but facilitates NPR1-NPR3 interaction. In the absence of SA, NPR4-mediated NPR1 degradation prevents NPR1 accumulation, whereas high SA levels also prevent NPR1 accumulation due to NPR3 [20].

Genes involved in the oxidative burst and anti-oxidation are activated in response to biotic and abiotic stresses, with *GOX* (glycolate oxidase) and *SAGT* (serine/alanine glyoxylate aminotransferase) playing an essential role in SA signaling. Excessive reactive oxygen species (ROS) cause dramatic changes in plant metabolism, even cell death. For self-protection, a set of antioxidant genes are actively induced to scavenge or quench ROS in maintaining redox homeostasis in plant cells. Peroxidase (POD), glutathione-S-transferase (GST), and glutathione reductase (GR) are powerful and effective antioxidants in plants. Under physiological conditions, glutathione exists in two forms: the oxidative GS (GSSG) and the reduced GS (GSH). The function of GR is to convert GSSG to GSH, which is the most abundant antioxidant, with approximately 90% of the total GS in cells [43]. Finally, genes coding for PR proteins, particularly PR1, lead to HR at the infection site and SAR in the distal part of the plant. Together, these genes form a complex network that can be considered as a candidate target for transcriptional detection.

### 1.3. Long-Distance Transport of SA and NHP Signals

SAR is a phenomenon that enhances disease resistance in plants by activating local defense mechanisms. Recent research by Lim et al. (2020) has revealed that the cuticle plays a crucial role in regulating the active transport of SA from infected parts of the plant to uninfected areas via the apoplast (space between cell wall and plasma membrane) [44]. Pathogen infection leads to the accumulation of SA in the apoplastic compartment, driven by a pH gradient. Under physiological pH, SA is preferentially partitioned into the apoplast and cuticular wax, rather than the symplast. The distal transport of SA is also regulated by water potential and transpiration [45]. These findings suggest that SA is a long-distance mobile signal that is vital for inducing SAR [44]. Recent studies have also demonstrated that NHP is a mobile defense signal that can induce SAR in plants [19,28,29,46,47].

Based on these discoveries, we propose ‘transcription-aided selection (TAS)’ for crop disease resistance. In fact, a large number of studies have shown that metabolites (such as amino acids, hormones, etc.) and disease resistance genes (such as R genes) can serve as unique markers of plant responses to abiotic and biotic stresses, and can be used to design new biotechnological or breeding strategies to enhance the relevant resistance of plants [48,49]. In this paper, we suggest that the transcription levels of genes can also be considered as part of this unique marker, and we name this TAS strategy. This strategy involves utilizing the positive regulatory roles of genes involved in plant immunity to screen germplasms with high disease resistance. The objective of this study is to validate the TAS strategy by providing evidence for its effectiveness.

## 2. Results

To prove the TAS strategy, we have conducted a series of experiments in which the transcriptional expression of *GmSAGT1* in 15 soybean varieties with SDM resistance ranked from HS to HR were analyzed. In addition, the RNA-seq of HR variety Jilinxiaoli 1 (JLXL 1) and HS variety Heinong 10 (HN 10) 72 h after inoculation with *Peronospora manshurica*, the cause agent of SDM, are performed for profiling differential gene expression. Furthermore, based on the differentially expressed genes (DEGs) data, the expression analysis of several target genes was conducted using RT-PCR, and the *GmSAGT1* and *GmPR1* were used as target genes for screening of 2501 soybean germplasms derived from China National GenBank for SDM resistance. Finally, 23 varieties were inoculated with soybean downy mildew in a greenhouse to analyze the consistency of the results obtained by TAS and pathogen inoculation, further verifying the effectiveness of the TAS strategy.

### 2.1. Correlation Analysis of GmSAGT1 Transcription and SDM Resistance

SA is the best-known signal, proven to be essential for the induction of SAR in most plants when they are invaded by pathogens. Similar to infection by *Peronospora manschurica*, we have found SA treatment also significantly increased the level of *GmSAGT1* expression with a 13 times higher induction in HR variety than that in HS, indicating it is feasible to use this technique to differentiate whether a soybean variety is HR or HS [50].

**Figure 1:**
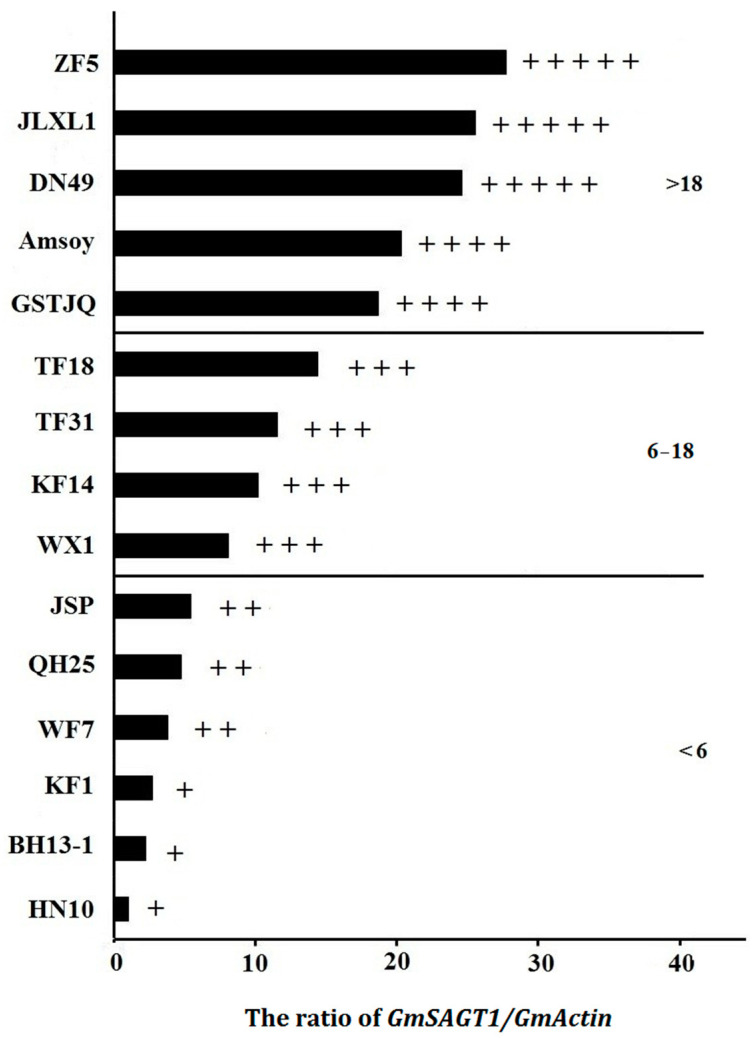
The correlation of SDM resistance and *GmSAGT1/GmActin* expression. ZF 5: Zaofeng 5; JLXL 1: Jilinxiaoli 1; DN 49: Dongnong 49; AS: Amsoy; GSTJQ: Guanshuitiejiaqing; TF 18: Tiefeng 18; TF 31: Tiefeng 31; KF 14: Kefeng 14; WX 1: Wuxing 1; JSP: Jingshanpu; QH 25: Qihuang 25; WF 7: Wenfeng 7; KF 1: Kefeng 1; BH 13-1: Binhai 13-1; HN 10: Heinong 10. +++++ represents highly resistant (HR) to soybean downy mildew (SDM) by either in vitro leaf/*Pm* assay or by field *Pm* inoculation in two consecutive years; ++++ represents resistant (R) to SDM; +++ moderate resistant (MR); ++ susceptible (S); + highly susceptible (HS). Note: (1) The *GmActin* in soybean genome is used as an internal reference; (2) the correlation coefficient R^2^ = 0.7981; (3) The SDM response is ranked five groups of HR, R, MR, S, and HS based on the data of the qRT-PCR assay, which is statistically different between each group at a level of *p* < 0.01 or *p* < 0.05 using a *t*-test.

Quantitative RT-PCR assay of 15 commercial soybean varieties in this study revealed that the average expression level of *GmSAGT1* in five groups of HR, R, MR, S, and HS were sequentially ranked from high to low (Figure 1). These data are consistent with the SDM resistance published in existing studies and the results obtained by either in vitro leaf/*Pm* assay or by field *Pm* inoculation in two consecutive years collaborated with colleagues in the Plant Protection College, Shenyang Agriculture University, China. In the qRT-PCR assay, the *GmActin* gene was used as an internal reference. The ratio of *GmSAGT1*/*GmActin* obtained by qRT-PCR is highly correlated with SDM resistance after *Pm* inoculation with a correlation coefficient R^2^ = 0.7981 (Figure 1). As a result, the 15 varieties can be categorized into three groups (HR, R/MR, and S/HS, respectively), demonstrating that the feasibility and effectiveness of TAS are validated for the SDM resistance ranking. Silverman et al. (1995) also reported that in their study of 28 rice varieties, the basic levels of SA were positively correlated with its resistance to rice blast with a coefficient of 0.477 [51].

### 2.2. Identifying and Selecting Hub Genes for TAS Technology Using RNA-Seq and RT-PCR

**Figure 2:**
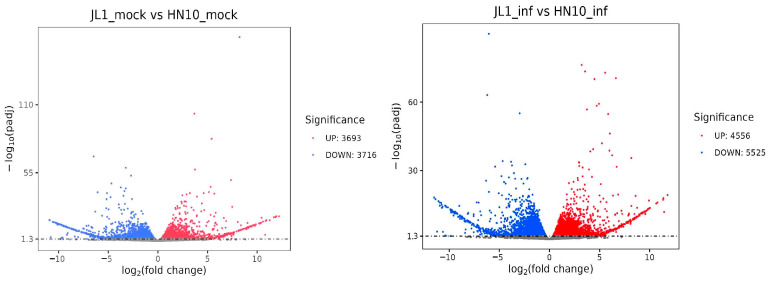
Volcano bolts of differentially expressed genes (DEG). The horizontal axis represents the fold change in gene expression across different samples, while the vertical axis indicates the statistical significance of the differences in gene expression levels. Genes with significant differential expression are represented by red dots (upregulated) and blue dots (downregulated).

**Figure 3:**
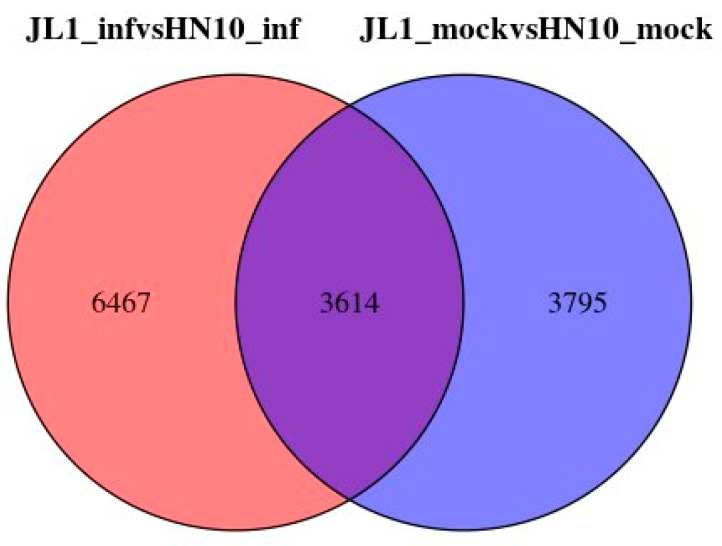
Venn diagram of differential genes. Pink represents the differential genes between JLXL 1 and HN 10 after inoculation, while blue represents the differential genes between JLXL 1 and HN 10 without inoculation. The overlapping section indicates the common differential genes.

We conducted transcriptomic sequencing to identify more differentially expressed genes from highly resistant and highly susceptible varieties, using the highly resistant variety JLXL 1 and the susceptible variety HN 10 as materials. We analyzed the differentially expressed genes in the leaves before and after inoculation with Pm for 72 h. A comparison between JLXL 1 (uninfected with *Pm*) and HN 10 (uninfected with *Pm*) revealed 7409 DEGs with a *p* value < 0.05 and log2 fold change >2 or <−2. JLXL 1 (infected with *Pm*) compared to HN 10 (infected with *Pm*) showed 10,081 DEGs. Adding both, there are a total of 17,489 DEGs (Figure 2). A comparison of duplicates found that among the 17,489 DEGs, 3614 were duplicates (Figure 3). Further analysis of these 3614 DEGs revealed that 18 DEGs were significantly expressed at higher levels in JLXL 1 than in HN 10 before and after *Pm* inoculation (Table 1).

Based on the transcriptome sequencing result and our previous article [52], the 18 DEGs, along with *GmSAGT1*, were further utilized in the RT-PCR assay. The ratio of *GmSAGT1*/*GmActin* in HR and HS was 14.83 and 0.47, while the ratio of *GmPR1*/*GmActin* for HR and HS was 0.61 and 0.02, respectively. The expression level of the rest of the genes in HR and HS was either no difference or too low to be detected. Results indicated that the expression level of *GmSAGT1* and *GmPR1* could be used as hub targets to differentiate HR and HS soybean collections (Table 2). Subsequently, the *GmSAGT1* and *GmPR1* are used in the screening of 2501 soybean germplasms for SDM resistance.

### 2.3. Screening Soybean Germplasms Using the TAS Strategy

**Figure 4:**
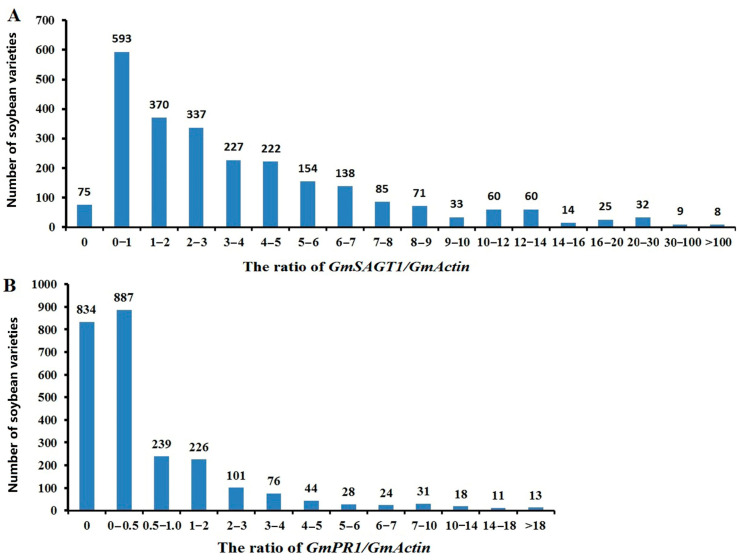
Analysis of the *GmSAGT1*/*GmActin* (**A**) and *GmPR1*/*GmActin* (**B**) ratios in 2501 soybean lines using RT-PCR assay. Most lines exhibit expression levels categorized as S/HS, following a skewed distribution pattern.

**Figure 5:**
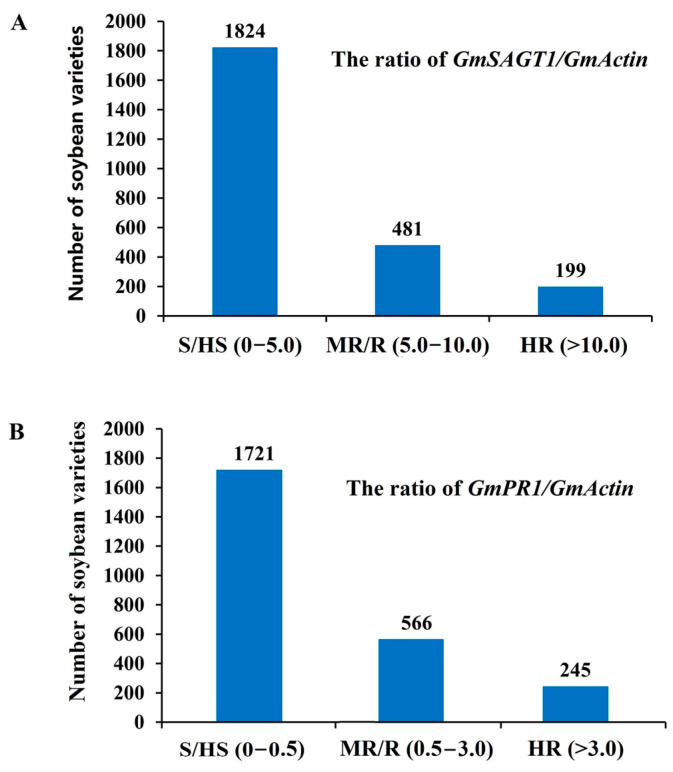
Number of soybean lines ranked as S/HS, MR/R, and HR based on the ratio of *GmSAGT1/GmActin* and *GmPR1/GmActin* using RT-PCR assay. (**A**): The ratio of *GmSAGT1/GmActin* 0–5, 5–10, and >10 is considered S/HS, MR/R, and HR, respectively. (**B**): The ratio of *GmPR1/GmActin* 0–0.5, 0.5–3, and >3 is considered S/HS, MR/R, and HR, respectively.

A total of 2501 soybean germplasms, including commercial cultivars, landraces, PI introductions, and wild soybean species, provided by the China National GenBank, were used for screening SDM resistance using RT-PCR detection of *GmSAGT1* and *GmPR1* expression. Results are presented in Figure 4, indicating that the expression level of majority lines is grouped as S/HS, with a pattern of skewed distribution (Figure 4). For ranking the expression level of the two genes, we have used the ratio of *GmSAGT1*/*GmActin* value of 0–5 as HS/S, 5–10 as MR/R, and >10 as HR, while it is 0–0.5, 0.5–3, and >3 for *PR1*, respectively. There are 1824 soybean varieties with a *GmSAGT1*/*GmActin* value between 0 and 5, 481 varieties with a value between 5 and 10, and 199 varieties with a value greater than 10. Additionally, there are 1721 soybean varieties with a *GmPR1*/*GmActin* value between 0 and 0.5, 566 varieties with a value between 0.5 and 3, and 245 varieties with a value greater than 245 (Figure 5). Among all soybean varieties, the proportion of *GmSAGT*1/*GmActin* values that are HR is 7.95%, and the proportion of *GmPR1/GmActin* values that are HR is 9.68%.

**Figure 6:**
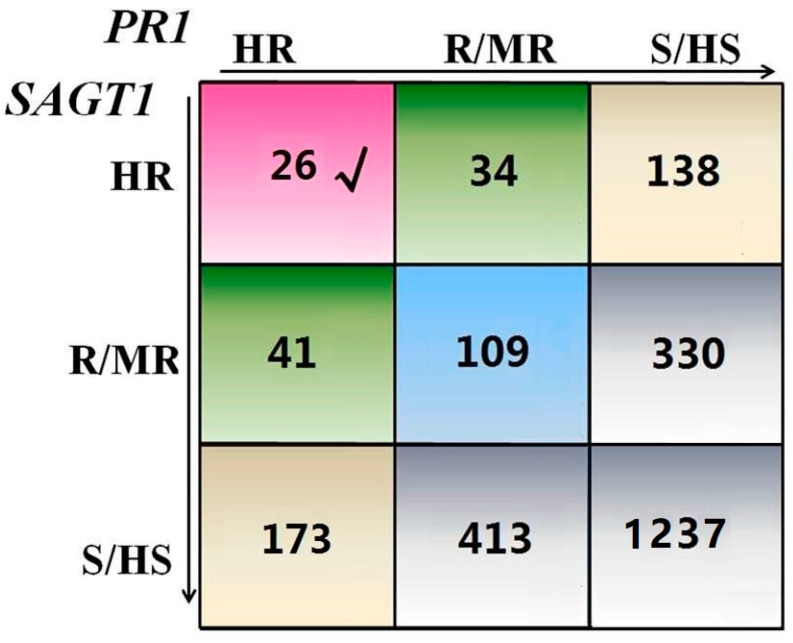
Soybean germplasms tested in five categories based on the expression level of *GmSAGT1* and *GmPR1* genes. The ratio of *GmSAGT1*/*GmActin* at a value of 0–5, 5–10, and >10 was considered as HS/S, MR/R, and HR, while it was 0–0.5, 0.5–3, and >3 for *GmPR1*/*GmActin*, respectively. Individual lines with detected values of the ratio were plotted into five categories of HR-HR (pink), HR-R/MR (green), R/MR-R/MR (blue), HR-S/HS (light brown), R/MR-S/HS and S/HS-S/HS (light gray) respectively. Numbers in each square are the number of lines in a given category. √: The 26 most significant varieties showing higher expression both *GmSAGT1* and *GmPR1* genes (HR-HR).

As a result, the data of *GmSAGT1* and *GmPR1* expression of the individual lines are plotted into a nine square grid to group lines into five categories of HR-HR, HR-R/MR, R/MR-R/MR, HR-S/HS, R/MR-S/HS and S/HS-S/HS (Figure 6). Of the total 2501 lines tested, 26 (~1%) exhibiting higher expression of both *GmSAGT1* and *GmPR1* gene (HR-HR) were selected that might be potentially considered as SDM-resistant resources for breeding. Upon conducting a literature search, we have discovered that six of these lines have been previously ranked for disease resistance (Table 3). Our findings align with previous reports, indicating that these materials possess broad-spectrum resistance to various soybean diseases such as SDM, FLS (frogeye leaf spot, *Cercospora sojina* Hara), SBB (soybean bacterial blight, *Pseudomonas syringae* pv. *glycinea*), SBS (soybean brown spot, *Septoria glycines* Hemmi), SBP (soybean bacterial pustule, *Xanthomonas phaseoli* var. *sojense*), and SMV (soybean mosaic virus). These facts demonstrate that it is possible to select resources with broad-spectrum resistance to different pathogens using the TAS strategy.

### 2.4. SDM Resistance Identification of Soybean Varieties Screened by TAS Strategy

To validate the SDM resistance of 26 varieties identified with high expression of both *GmSAGT1* and *GmPR1* genes (HR-HR) through TAS screening, all were initially selected for inoculation with *P. manshurica* in a controlled greenhouse environment. Subsequently, three varieties were excluded from the inoculation process due to low germination rates or unfavorable growth conditions, leaving 23 varieties for the inoculation experiment. Zaofeng 5 and Jiunong 9, known for high resistance, and Jiyu 120 and Heinong 10, known for high susceptibility, were used as controls. A total of three biological replicates were performed. To strictly differentiate SDM resistance, disease levels for each strain were determined using the maximum value of DI in three replicates rather than the average value of DI in the three replicates.

**Figure 7:**
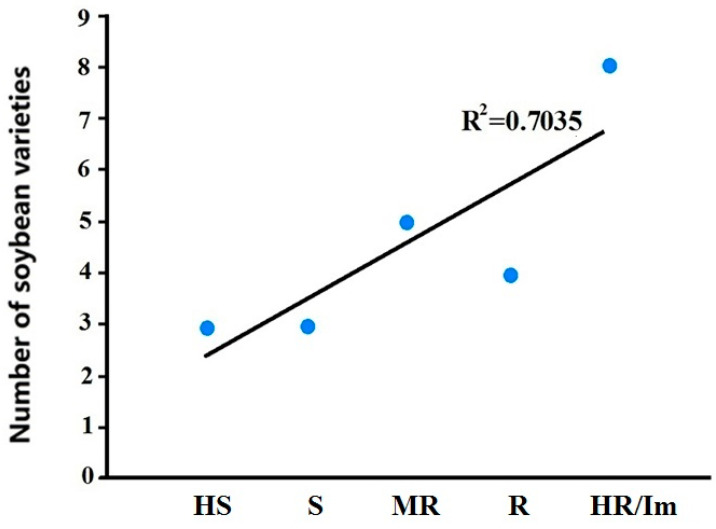
Resistance identification results of 23 soybean lines obtained after *Pm* inoculation. Soybean varieties with high expression of *GmSAGT1* and *GmPR1* were classified after *Pm* inoculation, with eight HR, four R, five MR, three S, and three HS.

**Figure 8:**
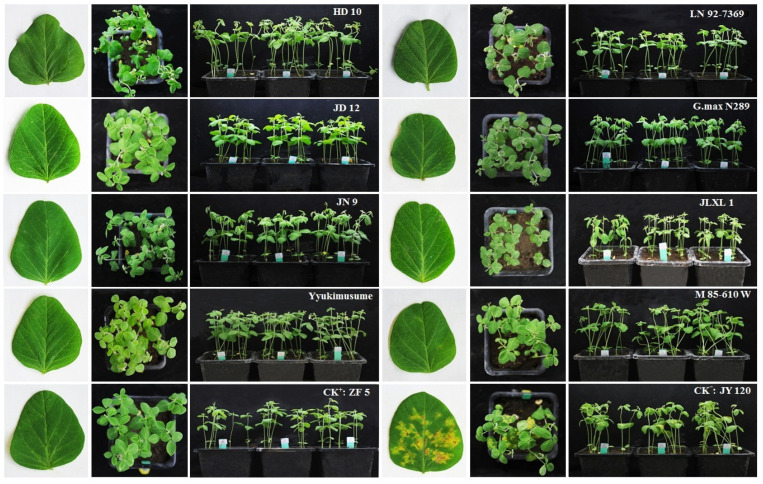
Eight soybean varieties showed high resistance or immunity to soybean powdery mildew 14 days after being inoculated with *Pm*. HD 10: Huaidou 10; JD 12: Jidou 12; JN 9: Jiunong 9; JLXL 1: Jilinxiaoli 1. ZF 5: Zaofeng 5; JY 120: JiYu 120. All collections were individually seeded in square pots with three biological replicates, and Sporangium suspensions were evenly applied to the back of soybean leaves (non-cotyledon). Disease levels were assessed 14 days after inoculation. ZF 5 was the positive control, and JY 120 was the negative control.

Based on the inoculation results of *P. manshurica*, out of twenty-three lines, eight showed HR (including seven immune), four were R, five were MR, three were S, and three were HS. The proportion of resistant lines (HR, R, and MR combined) reached 73.91%. The correlation coefficient R^2^ between TAS results and downy mildew inoculation results was 0.7035, as depicted in Figure 7. This suggests that the TAS strategy and the traditional resistance identification through *P. manshurica* inoculation are in good agreement, and TAS strategy can preliminarily identify highly resistant materials during the extensive screening of germplasm resources. The molecular analysis data and resistance identification by *Pm* inoculation, along with the disease resistance level of 23 materials, are presented in Table 4. After 14 days of *Pm* inoculation, please refer to Figure 8 for the status of the eight HR soybean varieties.

## 3. Discussion

TAS is founded on the understanding that resistance to plant pathogens is a complex trait governed by multiple genes. The expression of these genes is regulated by signal transduction pathways that are activated upon pathogen attack. Therefore, the transcriptomic analysis of plants in response to pathogen infection can provide valuable information on the genes involved in the immune response. By using this information, it is possible to identify the key genes that are responsible for disease resistance and use them as targets for transcriptional detection.

The TAS strategy involves four steps. (1) Conduct transcriptomic analysis of plants in response to pathogen infection to identify DEGs before and after pathogen infection between high-resistance and high-susceptibility varieties. (2) Analyze the transcription expression of DEGs in various disease-resistant varieties (from HR to HS), along with the correlation coefficient R^2^ between DEGs transcription expression and disease resistance. This aims to clarify whether the transcription expression of DEGs aligns well with traditional resistance identification through pathogen inoculation and to determine if DEGs can serve as markers for the extensive screening of germplasm resources. (3) Develop a transcriptional detection method for the selected DEGs. (4) Screen plant germplasms or segregation populations for disease resistance based on the expression level of the selected DEGs. Theoretically, the TAS strategy is applicable to any plant species and pathogens where the resistance is controlled by multiple genes and the signaling pathway in the whole net is clearly defined. The most critical issue is to select and define the most appropriate hub targets whose expression level may represent the level of resistance in a given pair of host–pathogen interactions.

### 3.1. Evidence for TAS Proving

#### 3.1.1. *SAGT1* Transcription Analysis

The SAGT is a bi-functional enzyme with both SGT and AGT activities, while the *GOX* is an upstream gene of the *SGT/AGT* in photorespiration. The function of GOX is to convert glycolic acid into glyoxylate and H_2_O_2_ by oxidation [55]. The combination of GOX and SAGT may play an essential role in SA signaling, leading to more H_2_O_2_ accumulation, which in turn induces HR and SAR. It has been reported that the enzymes of SGT, AGT, and SAGT are localized in the peroxisome [50,56,57].

In our previous study, *E. coli* cells over-expressing GmSAGT1 protein dramatically increased the concentration of H_2_O_2_ to as high as 920 mM (5-fold over the control), which resulted in significant retardation of bacterial growth. Over-expression of the *GmSAGT1* gene in transgenic tobacco enhanced H_2_O_2_ accumulation (1.4-fold higher than that in wild type) and increased disease resistance to tobacco sore skin caused by *Rhizoctonia solani*. A comparison of HR and HS soybean varieties showed that the level of *SAGT1* gene expression in HR was 13 times higher than that in HS. Northern blot analysis also showed an 18.5-fold difference between HR and HS [50]. Interestingly, Taler et al. (2004) cloned two genes, *AT*1 and *AT*2, coding for serine glyoxylate aminotransferase (SGT), from a wild melon ecotype PI, which was resistant to melon downy mildew (MDM) caused by *Pseudoperonospora cubensi*s [58]. They found that the level of SGT activity was positively correlated with MDM resistance.

To understand the SDM resistance mechanism, we cloned *GmSAGT1* genes and their promoter sequences both from HR variety Zaofeng 5 (*GmSAGT1*) and HS Heinong 10 (designated as *GmSAGT1a* and *GmSAGT1b*). In a comparison of the cDNA and promoter sequences of *GmSAGT1* with its analogs of *GmSAGT1a* and *GmSAGT1b* cloned from HS, no significant differences were found. In the promoter region of *GmSAGT1*, it contained two TGAC motifs of the SA-responsive element (*as*-1). The two *as*-1 elements were also found in the promoter regions of the *GmSAGT1a* and *GmSAGT1b* (data unpublished). Together with their high homology of amino acid sequence (≥98%), it was suggested that the differences of HR and HS soybean in response to SA induction were attributed to the transcriptional regulation.

WRKY transcription factors (TFs) are one of the largest families of transcriptional regulators in plants, with 72 representatives in *Arabidopsis* and more than 100 members in rice, soybean, or popular [59]. WRKY TFs are important components of a plant signaling net that regulate many plant processes in response to biotic and abiotic stimuli. WRKYs contain the highly conserved amino acid sequence WRKYGQK that binds to the TTGAC(C/T) W-box *cis*-element in the promoter of their target genes [59,60]. Interestingly, a CTGACT box similar to the W-box TTGAC(C/T) of WRKYs is also presented in the promoter of *GmSAGT1*. Moreover, it overlaps with the *as*-1 element (TGAC), suggesting the CTGACT box in *GmSAGT1* may interact with both GQK of W-box and SA. Indeed, we have found that the GmWRKY31 interacts with the *GmSAGT1* in yeast one-hybrid (Y1H) assay [60].

#### 3.1.2. Transcriptomic Analysis

With the advent of next-generation sequencing technology, the Illumina sequencing approach has been used for understanding the complexity of gene expression and regulation networks in plant response to different pathogen attacks. In soybeans, Kim et al. (2011, 2015) first reported the application of RNA-seq for profiling gene expression in soybeans in response to bacterial leaf pustule (*Xanthomonas axonopodis pv. glycines*) attack [61,62]. In the following years, transcriptomic analyses have been reported on Asian soybean rust (*Phakopsora pachyrhizi*) [63], soybean root and stem rot (*Phytophthora sojae*) [64], soybean downy mildew (*Peronospora manschurica*) [52,65], and soybean cyst nematode (SCN, *Heterodera glycines*) in common bean [66]. The responsive genes and metabolic pathways associated with disease resistance have been identified. The question is how to use this massive amount of data and incorporate it into breeding programs.

To answer this question, we have used healthy and *Pm*-inoculated leaves (72 hai) of HR Jilinxiaoli 1 (JLXL 1) and HS Heinong 10 (HN 10) as plant materials for RNA-seq. Results (Table 1) show that the basic expression of EDGs is all differentially expressed and highly induced by *Pm* inoculation in HR and HS varieties.

Based on the RT-PCR results (Table 2), we have selected *GmSAGT1* and *GmPR1* as hub targets because the *GmSAGT1* is highly involved in hypersensitive reaction on the infection site resulting in programmatic cell death, and there is a high correlation between the level of *GmSAGT1* expression and the SDM resistance. The *GmPR1* is an important pathogenesis-related protein highly involved in SAR induction.

#### 3.1.3. Screening Soybean Germplasms

The TAS strategy has been successfully applied to the screening of soybean germplasms for SDM resistance by using *GmSAGT1* and *GmPR1* for transcriptional detection. Results show that the selected 26 collections are promising candidates for further breeding.

Taken together, it is reasonable to suggest that the TAS can be applied not only for germplasm screening but also be applicable to design specific parental lines for crossing, aiming at pyramiding the number of resistant genes in the same elite variety and selecting individuals in segregated populations. In addition, it may also be valuable for evaluating disease resistance of advanced lines and varieties newly developed, as well as the cultivars currently used in commercial production.

#### 3.1.4. TAS Strategy Versus Traditional Pm Inoculation Identification

A total of 2501 soybean germplasms underwent hub gene expression detection, and 26 HR/HR lines were initially selected. Among them, 23 were subjected to Pm inoculation. Results showed that 17 had resistance levels at or above the medium-resistant level (accounting for 73.91%), with 7 reaching the immune level. Among these 7, 2 (Jiunong 9 and Jilinxiaoli 1) were previously known as highly resistant lines, while 5 (Huaidou 10, Jidou 12, Yyukimusume, LN92-7369, G.maxN289) were newly discovered lines that are immune to SDM. This indicates that the TAS strategy developed in this study can effectively select highly resistant varieties from a large amount of germplasm resources. Considering that the SA signal pathway can control resistance to specialized and facultative parasites, from a theoretical analysis, in addition to being resistant to SDM, these seven immune lines may also have the ability to resist other specialized and facultative parasites. Therefore, it is worthwhile to pay attention to and investigate these lines in future inoculation and field identification.

However, it must be pointed out that the above are still preliminary results. Differences in seed purity, seedling growth, and physiological condition, robustness, environmental conditions, and experimental operation details may lead to experimental errors or even misjudgment of disease severity. For example, in the hub gene expression level detection, the *GmSAGT1/GmActin* expression value of LN92-7369 was 24.89 ± 29.34, with a coefficient of variation of 117.88%. In the *P. manshurica* inoculation experiment, the DI (%) value of Ha 53 was 10.49 ± 15.08, with a coefficient of variation of 143.78%. The DI (%) values of the two susceptible controls, Heinong 10 and Jiyu 120, were 33.97 ± 27.34 and 66.58 ± 28.92, with coefficients of variation of 80.51% and 40.43% respectively. For caution, we will repeat the hub genes expression detection and *Pm* inoculation identification on seven immune materials and one HR material.

#### 3.1.5. Advantages of the TAS

Maker-aided selection (MAS) has been widely used in diverse crop breeding programs, and great progress has been made to date. When comparing TAS with MAS, we believe that the TAS is a step further. While MAS focuses on a piece of DNA sequence on the chromosome, the TAS displays gene expression in the whole SA signal net. The selected targets for detection are the key DEGs in the network and are all positively regulated by SA. Moreover, their expression levels are much higher in HR over HS (log_2_ fold change > 2.0). Thus, by detecting the expression of target gene(s), the differences between HR and HS will be displayed at the molecular level.

Briefly, the TAS has four advantages. (1) Through the detection of transcriptional expression of hub target genes in SA signal transduction, it is able to integrate positive roles of genes involved in plant immunity. It may reduce the complexity of the whole signal net and avoid the need to clarify diverse gene functions one by one. This simplification may achieve maximum results with little effort. In this sense, the TAS can be viewed as a “Simplified Precise Selection”. (2) TAS can be effectively used in massive screening of large quantities of crop germplasms and segregation populations to identify elite ones with broad-spectrum and persistent disease resistance. As a result, it saves land, labor, time, and cost. (3) The TAS may build up a link between basic and applied research to achieve a practical goal that will facilitate the massive data obtained from transcriptomic sequencing to be used in plant breeding practice. Manipulation at the molecular level would thus be possible. (4) In the entire TAS process, there is no molecular manipulation involved for gene insertion, deletion, or knockout. The only thing needed is to detect target gene expression. Therefore, it will be out of the scope of GMO regulation to facilitate the commercialization of breeding products.

TAS has the advantages mentioned above, but high gene expression does not directly imply a significant improvement in metabolic pathways. Proteins are key to executing functions and can be regulated in various ways. Therefore, it should be verified in the future whether the protein levels corresponding to those genes with high expression levels in the germplasms have also increased and analyzed their relationship with plant disease resistance.

## 4. Materials and Methods

### 4.1. GmSAGT1 Transcriptional Expression and SDM Resistance

Fifteen soybean varieties ranked as HR, R, MR, S, and HS to SDM were used in this study. The top third expanded leaves of 40-day-old plants were sampled from at least three plants for each variety and then mixed as one sample for the qRT-PCR assay of *GmSAGT1* expression with three biological replicates. The methods of RNA extraction, reverse transcription into cDNA, and quantitative RT-PCR (three technical replicates)were as described previously [50]. To confirm SDM resistance or susceptibility of individual variety, parallel experiments were conducted by field Pm inoculation of 15 varieties in two consecutive years, collaborated with colleagues in the Plant Protection College, Shenyang Agriculture University, China. Finally, the correlation coefficient between the level of *GmSAGT1* expression and the SDM resistance was calculated. The specific method was described in our previous paper [52].

### 4.2. RNA-Seq of Pm-Inoculated SDM-HR and -HS Soybean Variety

Plants of SDM-HR variety JLXL 1 and HS HN 10 were grown in a greenhouse. The back side of the third expanded leaf at the top of the plant with an age of 14 days was brushed with an inoculant of the pathogenic fungus using a brush (*Pm*-inoculated), while distilled water was used as a control. After 72 h of inoculation and moisturizing, samples were collected, and total RNA was extracted and reverse transcribed into cDNA. RNA-seq sequencing was performed by the Allwegene Co., Nanjing, China, to screen for DEGs related to SDM resistance.

### 4.3. Screening Soybean Germplasms by RT-PCR

A total of 2501 soybean germplasms were provided by the China National Crop GenBank, Beijing, China. Seeds were sown in nutrient bowls and placed in a light incubator with a 16 h light/8 h dark cycle at 25 °C. Soybean cotyledon samples were collected 7–8 days after sowing and frozen in liquid nitrogen. Six cotyledons were taken from each line and mixed to reduce errors caused by individual differences.

Total RNA was extracted using the RNAprep Pure Plant Kit (Tiangene Co., Beijing, China) according to the manufacturer’s instructions. The quality of total RNA was assessed using a Nanodrop spectrophotometer (Thermo Fisher Scientific Inc., Waltham, MA, USA) and reverse transcribed into cDNA using the HiScript III 1st Strand cDNA Synthesis Kit (Vazyme Co., Nanjing, China).

The transcription levels of two genes, *GmSAGT1* and *GmPR1*, were detected using RT-PCR with three technical replicates. Based on our cloned gene sequences, primers were designed for *GmSAGT1* (Glyma08G41460): *GmSAGT1*/F: CCATTTCTCATCCCTACAAC and *GmSAGT1*/R: CAACCACAATACCTATCCC. According to RNA-seq data, the *GmPR1* primers were designed based on the conserved regions of the two *GmPR1* genes (Glyma15G06780 and Glyma15G06790): *GmPR1*/F: AATGCACACAACGCTGC and *GmPR1*/R: TACTTTGGCACATCCAAG. *GmActin* (LOC100781142) was used as an internal reference gene to calculate the ratio of target gene/*GmActin*, and its primers were designed as follows: *GmActin*/F: GCGTGATCTCACTGATGCC and *GmActin*/R: TCGCAATCCACATCTGTTGG. The RT-PCR protocol for *GmSAGT1* and *GmActin* included an initial pre-denaturation step at 95 °C for 5 min, followed by 23 cycles of 95 °C for 15 s, 56 °C for 30 s, and 72 °C for 15 s, with a final extension at 72 °C for 5 min. For *GmPR1* and *GmActin*, the RT-PCR reaction conditions included pre-denaturation at 95 °C for 5 min, followed by 24 cycles of 95 °C for 15 s, 53 °C for 30 s, and 72 °C for 15 s, with a final extension at 72 °C for 5 min. The RT-PCR products of *GmSAGT1* and *GmPR1* were detected using 1.5% agarose gel electrophoresis at 150 V for 40 min and then imaged using a gel imaging system. The Image J 1.53k software was used to scan and quantify the ratios of *GmSAGT1*/*GmActin* and *GmPR1*/*GmActin*.

### 4.4. Inoculation of Peronospora Manshurica for Identification of Soybean Germplasm Resistance to Downy Mildew Disease

The soybean varieties used for *Pm* inoculation included twenty-three HR/HR materials, two highly resistant control materials (Zaofeng 5 and Jiunong 9), and two susceptible control materials (Jiyu 120 and Heinong 10). The above materials were sown in square flowerpots and grown under conditions of 16 h of light/8 h of darkness at a temperature of 18–23 °C until the first pair of true leaves was fully expanded. Weak seedlings were removed, leaving 12 healthy seedlings for each material (3 biological replicates). Following the method of Sun et al. (2015) [50], a suspension of *Pm* spores (concentration of 1 × 10^5^) was prepared and evenly spread on the back of the first pair of true leaves of the soybean. After inoculation, the plants were transferred to a controlled environment chamber for humid cultivation (temperature 18–23 °C, humidity 100%). After 14 days, the disease severity was measured using a standard disease scale (0–9) (0 = no chlorosis or other signs of infection to 9 = lesions account for more than 51% of the leaf area), as described in Table 5. The disease severity score was converted to a disease index (DI) using the formula: disease index (DI) = ([0 × n0 + 1 × n1 + 3 × n3 + 5 × n5 + 7 × n7 + 9 × n9]/(9 × N) × 100, where n_i_ (i = 0 to 9) represents number of plants in each corresponding disease severity score categories of 0 to 9, respectively, and N is the total number of plants assessed. The resistance level of the soybean germplasms was determined based on the maximum DI value from the three biological replicates (Table 6).

## 5. Conclusions

It is well known that pyramiding multiple genes into the same cultivar is an effective strategy to enhance the durability of the resistance. TAS may play an essential role in the implementation of such a strategy.

Due to the complexity of the regulatory network, the most crucial issue is to define the hub target gene(s) to be detected for a given pair of host–pathogen interactions. For example, we have also defined four genes as targets in screening soybean cyst nematode (SCN) resistant resources in the Chinese soybean landraces. The results will be published elsewhere. In addition, the detection techniques also need to be optimized and standardized for each pair of crop–pathogen, including the clarification criteria for grouping or ranking the disease resistance by RT-PCR data. All of these should be further defined one by one. In the research stage, parallelly detecting and comparing transcription levels of several target genes will avoid mis-ranking disease severity. However, in the sense of practical use, the techniques required would be as simple, easy, rapid, and efficient as possible so that it would be more convenient for breeder use. Furthermore, particular attention should be paid to the discovery of new resources in the closely related wild species that can be sexually crossed with cultivated crops. Consequently, the best lines with HR characteristics will be integrated into the breeding program, and TDB technology will eventually be developed. To expand the applications of TAS, it is reasonable to speculate that the search for appropriate target genes in plant hormone signaling in response to abiotic stress, such as salinity and drought, is likely to be able to select tolerant resources in the future.

## Figures and Tables

**Table 1:** Transcripts of DEGs in healthy and *Pm*-inoculated leaves of HR and HS soybean varieties using RNA-seq.

Gene	Gene ID	Read Counts		log_2_FC	Read Counts		log_2_FC
		JLXL 1 ni	HN 10 ni		JLXL 1 i	HN 10 i	
*GmPR1*	GLYMA_02G062400	51.69	13.29	1.97	274.87	16.71	4.03
*GmUreD*	GLYMA_02G163900	94.09	11.87	3.02	101.62	2.07	5.72
*GmST*	GLYMA_04G105200	116.99	29.88	1.97	442.69	29.12	3.91
*GmU-box12*	GLYMA_04G140100	115.57	13.32	3.13	247.06	6.77	5.12
*GmP21*	GLYMA_05G204800	255.90	0.99	8.05	310.74	0.40	9.55
*GmGsSRK*	GLYMA_06G258900	419.27	114.95	1.87	1025.78	76.57	3.74
*GmHCT*	GLYMA_07G021600	92.49	29.89	1.65	269.61	42.76	2.65
*GmHHT1*	GLYMA_08G010500	622.04	216.71	1.52	1209.04	176.48	2.77
*GmAP1*	GLYMA_11G064600	160.68	1.57	6.65	186.82	0.66	7.93
*GmRPP13*	GLYMA_13G187900	437.13	47.23	3.22	559.95	18.09	4.94
*GmDRP*	GLYMA_13G190800	271.38	7.77	5.14	351.86	4.56	6.26
*Gmβ-Glu12*	GLYMA_15G031400	1110.69	194.89	2.51	3038.39	147.80	4.36
*Gm31500*	GLYMA_15G031500	821.66	100.47	3.03	2302.02	48.33	5.56
*Gm71400*	GLYMA_16G071400	162.10	36.64	2.15	274.40	27.54	3.31
*GmCRK48*	GLYMA_16G202400	527.74	85.73	2.63	808.22	47.84	4.07
*GmMG13*	GLYMA_16G215100	81.64	1.87	5.62	166.60	1.16	7.18
*GmRPP4*	GLYMA_18G107000	162.87	2.81	5.82	221.54	1.86	6.86
*Gm50100*	GLYMA_20G050100	150.30	1.15	6.88	193.22	0.00	9.83

Note: JLXL 1: Jilinxiaoli1, highly resistant to soybean downy mildew (SDM). HN 10: Heinong10, highly susceptible to SDM. Read counts represent transcript abundance of the two genes tested. log_2_FC represents log_2_ fold change by comparison of the read counts of JLXL 1 and HN 10 samples. JLXL 1 ni and HN 10 ni: non-inoculated samples of JLXL 1 and HN 10; JLXL 1 i and HN 10 i: *Pm*-inoculated samples of JLXL 1 and HN 10.

**Table 2:** RT-PCR detection of candidate genes expression in HR and HS soybean varieties.

Gene	Gene ID	*GmTarget/GmActin*	
		JLXL 1	HN 10
*Gm* *PR1*	GLYMA_02G062400	0.61	0.02
*Gm* *UreD*	GLYMA_02G163900	0.07	0.01
*Gm* *ST*	GLYMA_04G105200	0.00	0.00
*Gm* *U-box12*	GLYMA_04G140100	0.00	0.00
*Gm* *P21*	GLYMA_05G204800	0.03	0.00
*Gm* *GsSRK*	GLYMA_06G258900	0.07	0.03
*Gm* *HCT*	GLYMA_07G021600	0.00	0.00
*Gm* *HHT1*	GLYMA_08G010500	0.15	0.06
*GmSAGT1*	GLYMA_08G302600	14.83	0.47
*Gm* *AP1*	GLYMA_11G064600	0.00	0.00
*Gm* *RPP13*	GLYMA_13G187900	0.06	0.00
*Gm* *DRP*	GLYMA_13G190800	0.07	0.01
*Gm* *β-Glu12*	GLYMA_15G031400	0.27	0.02
*Gm* *31500*	GLYMA_15G031500	0.02	0.00
*Gm* *71400*	GLYMA_16G071400	0.02	0.01
*Gm* *CRK48*	GLYMA_16G202400	0.02	0.01
*Gm* *MG13*	GLYMA_16G215100	0.04	0.00
*Gm* *RPP4*	GLYMA_18G107000	0.06	0.00
*Gm* *50100*	GLYMA_20G050100	0.02	0.00

**Table 3:** Comparison of disease ranking of six soybean lines in this study and previous studies.

Lines	*GmSAGT1/GmActin*	*GmPR1/GmActin*	Ranking by This Study	Ranking by Previous Study	Reference
JN 36	11.64 ± 0.99	6.88 ± 4.87	HR-HR	HR to SDM, FLS, SBB, and SMV, R to SBS	VAI
FS 26	10.02 ± 0.29	3.01 ± 0.06	HR-HR	R to FLS	[53]
Platte	18.29 ± 6.196	6.10 ± 1.01	HR-HR	R to SRR (races 1, 2, 10 and 16) and SBP	[54]
KJD 22	141.93 ± 39.54	9.56 ± 1.21	HR-HR	R to SMV SC3 strain; S to SMV SC7 Strain	VAI
JD 12	10.28 ± 0.33	4.42 ± 0.46	HR-HR	HR to SMV	VAI
WD 7	10.67 ± 2.11	4.48 ± 0.22	HR-HR	HR to SMV SC3 and SC7 strains	VAI

Note: JN 36: Jiunong 36; FS 26: Fengshou 26; KJD 22: Kenjiandou 22; JD 12: Jidou 12; WD 7: Weidou 7. Disease ranking: HR, highly resistant; R: resistant; S: susceptible. SDM: soybean downy mildew, caused by *Peronospora manshurica*; FLS: frogeye leaf spot, caused by *Cercospora sojina* Hara; SBB: soybean bacterial blight, caused by *Pseudomonas syringae pv. glycinea*; SMV: soybean mosaic virus; SBS: soybean brown spot, caused by *Septoria glycines* Hemmi; SRR: soybean root rot, caused by *Phytophthora sojae*; SBP: soybean bacterial pustule, caused by *Xanthomonas phaseoli var. sojense.* VAI: variety approval inquiry.

**Table 4:** The expression of *SAGT* and *PR1* in 23 soybean varieties and their resistance to SDM.

Varieties	Expression of *SAGT1* and *PR1*			DI and SDM Resistance After *Pm* Inoculation		
	*SAGT/Actin*	*PR1/Actin*	Rank	DI Average (%)	DI Max (%)	Resistance
HD 10	18.10 ± 0.10	3.16 ± 0.13	HR/HR	0.00 ± 0.00	0.00	Immune
JD 12	10.28 ± 0.33	4.42 ± 0.46	HR/HR	0.00 ± 0.00	0.00	Immune
JN 9	18.24 ± 1.82	0.57 ± 0.09	HR/HR	0.00 ± 0.00	0.00	Immune
Yyu	16.79 ± 8.46	7.31 ± 0.07	HR/HR	0.00 ± 0.00	0.00	Immune
LN 92	24.89 ± 29.34	6.26 ± 0.24	HR/HR	0.00 ± 0.00	0.00	Immune
GN 289	10.54 ± 0.88	5.22 ± 0.04	HR/HR	0.00 ± 0.00	0.00	Immune
JLXL 1	14.83 ± 2.62	0.61 ± 0.16	HR/HR	0.00 ± 0.00	0.00	Immune
M 85	13.52 ± 2.33	4.80 ± 0.43	HR/HR	8.33 ± 8.34	16.67	HR
XY 5	10.81 ± 0.35	4.18 ± 0.08	HR/HR	16.02 ± 8.77	24.24	R
Ha 53	10.07 ± 0.17	3.66 ± 0.14	HR/HR	10.49 ± 15.08	27.78	R
Л-234	13.07 ± 4.78	5.67 ± 2.13	HR/HR	27.34 ± 16.31	39.81	R
N 38	48.27 ± 14.17	6.76 ± 3.84	HR/HR	23.39 ± 21.18	39.81	R
YBT	14.65 ± 1.13	11.21 ± 0.45	HR/HR	36.43 ± 3.94	41.27	MR
Platte	18.29 ± 6.20	6.10 ± 1.01	HR/HR	36.48 ± 4.63	41.41	MR
HD 7	10.67 ± 2.11	4.48 ± 0.22	HR/HR	41.54 ± 12.30	52.78	MR
FS 26	10.02 ± 0.29	3.01 ± 0.06	HR/HR	48.43 ± 6.43	55.56	MR
MD 26	10.36 ± 0.38	9.44 ± 0.38	HR/HR	53.35 ± 5.13	59.26	MR
BN 7	10.56 ± 0.99	4.70 ± 0.11	HR/HR	59.88 ± 10.97	66.67	S
Soer-5	10.26 ± 0.22	5.37 ± 0.01	HR/HR	69.22 ± 6.99	75.56	S
QD 3	12.22 ± 0.19	3.15 ± 0.17	HR/HR	53.46 ± 27.69	77.78	S
JN 36	11.64 ± 0.99	6.88 ± 4.87	HR/HR	67.90 ± 28.26	87.88	HS
Bys 2	18.54 ± 0.83	4.57 ± 0.11	HR/HR	94.60 ± 2.49	96.83	HS
GX 3	11.60 ± 0.19	4.43 ± 0.09	HR/HR	75.93 ± 25.05	100.00	HS

Note: HD 10: Huaidou 10; JD 12: Jidou 12; JN 9: Jiunong 9; Yyu: Yyukimusume; LN 92: LN92-7369; GN 289: G.max N 289; JLXL 1: Jilinxiaoli 1; M 85: M85-610W; XY 5: Xinyin 5; YBT: Yuan-Bau-Tczin; HD 7: Huaidou 7; FS 26: Fengshou 26; MD 26: Mengdou 26; BN 7: Bainong 7; QD 3: Qiandou 3; JN 36: Jiunong 36; Bys 2: Bystritca 2; GX 3: Guixia 3. The level of disease in this identification was determined using the maximum value of DI in three replicates, which results in a more stringent classification of the level of disease.

**Table 5:** SDM disease severity score.

Disease Symptoms	Disease Severity Score
No chlorosis or other signs of infection	0
Only a few localized punctate lesions on the leaves, which account for less than 1% of leaf area	1
Irregular chlorotic lesions scattered on the leaves, lesions cover 1–5% of the leaf area	3
Lesions expanding, covering 6–20% of leaf area	5
Extended lesions, which account for 21% to 50% of the leaf area	7
Extended lesions, irregularly shaped large lesions covering more than 51% of leaf area	9

**Table 6:** Criteria for distinguishing SDM resistance level based on DI.

Disease Index (*DI*)	Resistance Level
0 ≤ DI ≤ 5	Immune
5 < DI ≤ 20	Highly resistant
20 < DI ≤ 40	Resistant
40 < DI ≤ 60	Moderately resistant
60 < DI ≤ 80	Susceptible
80 < DI ≤ 100	Highly susceptible

## Data Availability

The original contributions presented in the study are included in the article. Further inquiries can be directed to the corresponding authors.
